# Dual Topoisomerase I/II Inhibition-Induced Apoptosis and Necro-Apoptosis in Cancer Cells by a Novel Ciprofloxacin Derivative via RIPK1/RIPK3/MLKL Activation

**DOI:** 10.3390/molecules27227993

**Published:** 2022-11-17

**Authors:** Rania Alaaeldin, Islam M. Abdel-Rahman, Fares E. M. Ali, Amany Abdlrehim Bekhit, Eyad Y. Elhamadany, Qing-Li Zhao, Zheng-Guo Cui, Moustafa Fathy

**Affiliations:** 1 Department of Biochemistry, Faculty of Pharmacy, Deraya University, Minia 61111, Egypt; 2 Department of Pharmaceutical Chemistry, Faculty of Pharmacy, Deraya University, Minia 61111, Egypt; 3 Department of Pharmacology and Toxicology, Faculty of Pharmacy, Al-Azhar University, Assiut Branch, Assiut 71524, Egypt; 4 Department of Biochemistry, Faculty of Pharmacy, Minia University, Minia 61519, Egypt; 5 Innovative Research Center, Faculty of Pharmacy, Deraya University, Minia 61111, Egypt; 6 Department of Radiology, Graduate School of Medicine and Pharmaceutical Sciences, University of Toyama, Toyama 930-0194, Japan; 7 Department of Environmental Health, School of Medical Sciences, University of Fukui, Fukui 910-1193, Japan; 8 Department of Regenerative Medicine, Graduate School of Medicine and Pharmaceutical Sciences, University of Toyama, Toyama 930-0194, Japan

**Keywords:** ciprofloxacin derivative, HepG2, A549 cancer cells, apoptosis, necro-apoptosis, RIPK1/RIPK3/MLKL pathway, LDH, caspase 3

## Abstract

Fluoroquinolones (FQs) are synthetic broad-spectrum antimicrobial agents that have been recently repurposed to anticancer candidates. Designing new derivatives of FQs with different moieties to target DNA topoisomerases could improve their anticancer efficacy. The present study aimed to synthesize a novel ciprofloxacin derivative, examine its anticancer activity against HepG2 and A549 cancer cells, and investigate the possible molecular mechanism underlying this activity by examining its ability to inhibit the topo I/II activity and to induce the apoptotic and necro-apoptotic pathways. Molecular docking, cell viability, cell migration, colony formation, cell cycle, Annexin V, lactate dehydrogenase (LDH) release, ELISA, and western blotting assays were utilized. Molecular docking results showed that this novel ciprofloxacin derivative exerted dual topo I and topo II binding and inhibition. It significantly inhibited the proliferation of A549 and HepG2 cancer cells and decreased their cell migration and colony formation abilities. In addition, it significantly increased the % of apoptotic cells, caused cell cycle arrest at G2/M phase, and elevated the LDH release levels in both cancer cells. Furthermore, it increased the expression of cleaved caspase 3, RIPK1, RIPK3, and MLKL proteins. This novel ciprofloxacin derivative exerted substantial dual inhibition of topo I/II enzyme activities, showed antiproliferative activity, suppressed the cell migration and colony formation abilities for A549 and HepG2 cancer cells and activated the apoptotic pathway. In addition, it initiated another backup deadly pathway, necro-apoptosis, through the activation of the RIPK1/RIPK3/MLKL pathway.

## 1. Introduction

According to the World Health Organization, cancer is the second leading cause of death worldwide [1]. Cancer tissues consist of heterogenous cells with different characters leading to resistance to normal therapy and recurrence [2,3,4]. Today’s research focuses on developing new effective anticancer agents against several biological targets [5,6,7]. DNA topoisomerases (topo) are considered one of the crucial molecular targets for cancer therapy, owing to their essential role during DNA replication [8]. Topoisomerases are classified of topo I and II, whereas topo I cleaves a single DNA strand, and topo II cleaves both DNA strands. They are over-expressed in cancer cells. Thus, inhibition of topo I and II results in DNA damage with subsequent cancer cell death. Several anticancer drugs, including camptothecin and doxorubicin, are identified as topo I and II inhibitors, respectively [9]. Different pathways, like apoptosis and necro-apoptosis pathways, which are involved in the pathogenesis of different cancers, are affected by topoisomerase inhibitors [10].

Fluoroquinolones (FQs) are synthetic broad-spectrum antimicrobial agents that inhibit bacterial DNA synthesis through the inhibition of bacterial DNA gyrase and topo IV [11,12]. They are safe FDA-approved antibacterial agents that show no toxic activity on normal healthy cells at bactericidal doses. The structural similarities between bacterial DNA gyrase and human topo II attracted scientists to investigate the anticancer activities of FQs as a therapeutic approach in cancer treatment [13]. Ciprofloxacin is the most attractive member among FQs that has been extensively studied as an anticancer agent. It was reported that the bactericidal concentration of ciprofloxacin to exert its antimicrobial activity is 7μg/ml. However, to shift ciprofloxacin from an antibacterial agent to an anticancer agent, higher concentrations of ciprofloxacin are required to notably inhibit topo II with consequent cancer cell death, which could be harmful to normal healthy cells [14]. For example, the IC50 of ciprofloxacin was 388.6 μM against glioblastoma A-172 cell line, [15], and it was 160.4 μM ± 6.7, 200.4 μM ± 4.9, and 101.4 μM ± 3.6 for SW480, SW620, and PC3 cancel cells, respectively [16]. Moreover, the IC50 of ciprofloxacin against A549 and HepG2 cancer cells was 133.3 and 60.5 μg/ml, respectively [17]. Thus, several derivatives of ciprofloxacin have been synthesized and screened against many cancer cells to obtain new compounds with considerable anticancer activity at low concentrations and non-toxic activity on healthy normal cells [18,19].

The design of new ciprofloxacin derivatives is achieved through introducing different moieties at the *N*-4 position of the piperazine ring, which alter the physicochemical properties of the parent drug and improve the lipophilicity in addition to the cytotoxic efficacy [4]. The inclusion of Mannich bases is one of the practical modifications to ciprofloxacin for cancer therapy. For instance, Mannich bases of 1-naphthol and 8-hydroquinoline with piperidine and 4-arylsulfonylpiperazines achieved potent cytotoxic activity against a panel of carcinoma cell lines through the induction of apoptosis and activation of caspase-dependent pathways [20,21]. Additionally, phenolic Mannich bases were also reported with cytotoxic activities through topoisomerase inhibition [22,23].

Developing new agents with novel pharmacological applications became a valuable approach [24,25,26,27,28,29]. As an alternative strategy for cancer therapy, targeting the necro-apoptosis pathway has got a great attention [30].Therefore, the present study aimed to synthesize a novel C7-piperazinyl ciprofloxacin *ortho*-phenolic chalcone Mannich-base derivative (CM derivative) and examine its anticancer activity against different cancer cell lines, especially the hepatic cancer cell line (HepG2) and the non-small lung carcinoma cell line (A549). Furthermore, this study investigated the possible molecular mechanism underlying this effect by examining its ability to inhibit the topo I/II activity and to induce the apoptotic and necro-apoptotic pathways.

## 2. Results

### 2.1. Docking of the CM Derivative to Topo I and II Enzymes

**Figure 1:**
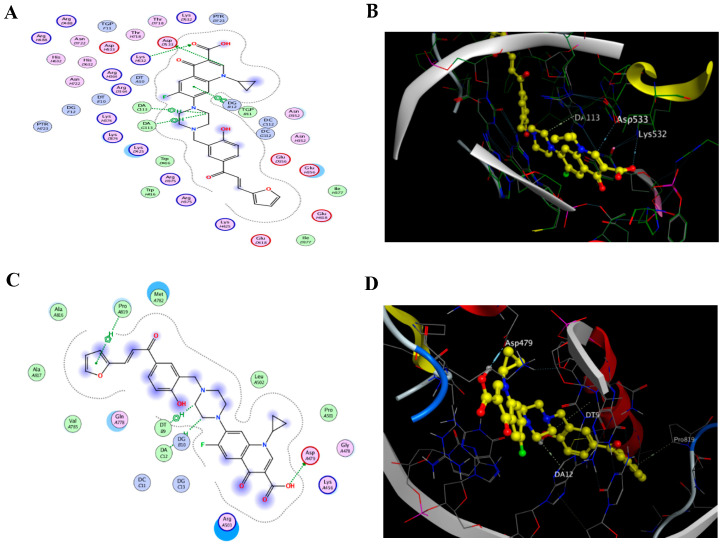
Docking to topo I and II enzymes. (**A**) 2D interactions and (**B**) 3D docking pose of the CM derivative in the active pocket site of the topo I enzyme (PDB code:1T8I), (**C**) 2D interactions and (**D**) 3D docking pose of the CM derivative in the active pocket site of the topo II enzyme (PDB code:3Qx3).

The docking results of camptothecin and the CM derivative, listed in Table 1, showed that the CM derivative acquired higher affinity towards binding with the protein rather than its attached DNA segment as it achieved an energy score of −11.605 kcal/mol, along with Hb interactions with Asp 533 amino acid residue as a hydrogen bond donor and another one with Lys 532 as a hydrogen bond acceptor and three hydrophobic interactions with (DA 113 and TGP 11) DNA nucleobases (Figure 1), which resembles the interactions pattern of the camptothecin.

Regarding the topo II B protein, the ligand (etoposide) was re-docked with the active pocket site and showed interactions of 2 H-bond interactions with Asp 479 and DG 13 DNA nucleotide base and only one H-pi interaction with nucleobase DA12; the docking algorithm was able to predict the co-crystalized ligand pose with least RMSD with an energy score of −7.671 kcal/mol. Meanwhile, docking results listed in (Table 2) displayed that the Ciprofloxacin Mannich base of the CM derivative has more tendency to bind with Topo II B enzyme as it shows a binding energy score of −8.247 Kcal/mol, much better than the one scored by the ligand etoposide.

### 2.2. Cell Viability

**Figure 2:**
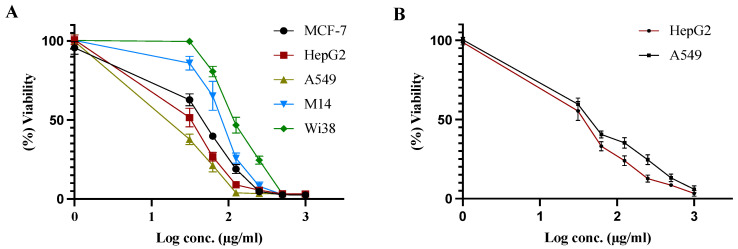
Cell viability assay (**A**). Effect of CM derivative on the viability of MCF-7, HepG2, A549, M14, and Wi38 cells. (**B**) Effect of etoposide on the of viability of HepG2 and A549 cancer cells. Viability was estimated before and after treatment for 48 h at different concentrations.

To evaluate the cytotoxic activity of the CM derivative, a cell viability assay was performed on different cancer cell lines, MCF-7, HepG2, A549, and M14, in addition to one normal human diploid cell line: Wi38. As shown in Figure 2A and Table 3, the CM derivative significantly decreased cell proliferation in MCF-7, HepG2, A549, and M14 cancer cells in a dose-dependent manner. The IC50 of the CM derivative against different cell lines was presented in Table 3. As shown, HepG2 and A549 cancer cell lines had the lower IC_50_s, therefore, they were chosen for further investigations in the present study.

Additionally, etoposide was used as a positive control in the present study against HepG2 and A549 cancer cell lines, as they exhibited the lower IC_50_s for the CM derivative. Etoposide significantly decreased the cell proliferation HepG2 and A549 cancer cells in a dose-dependent manner, as shown in Figure 2B.

### 2.3. Cell Migration Assay

**Figure 3:**
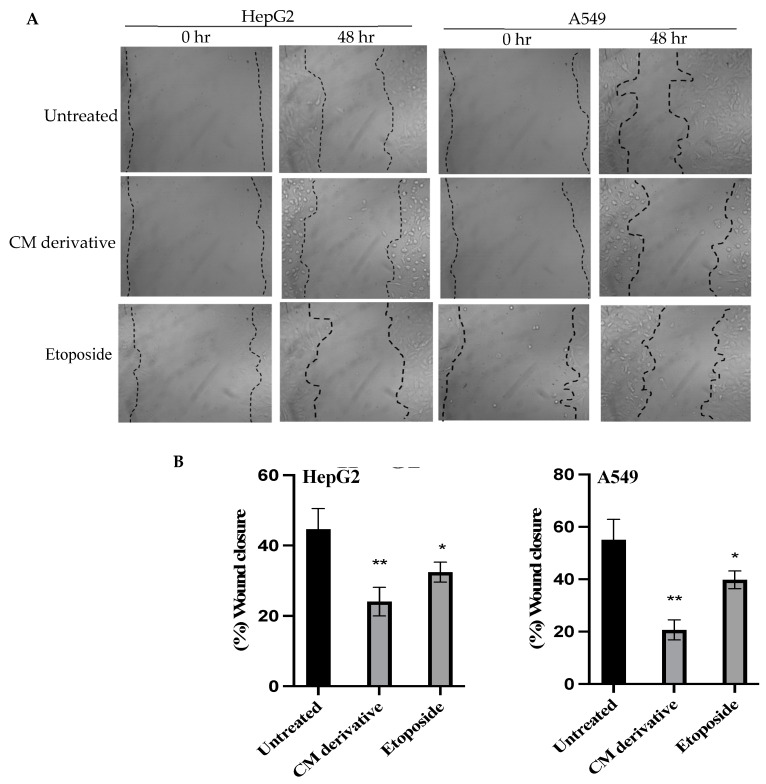
The effect of the CM derivative and etoposide on the migration capacity of cancer cells. (**A**) Representative photos were taken at 0 and 48 h for untreated and treated HepG2 and A549 cancer cells with the IC_50_ of CM derivative and etoposide after making the scratch area. *Magnification: x40*. (**B**) Percentage of wound closure for HepG2 and A549 cancer cells, treated with CM derivative and etoposide. Data represent mean ± SD. Significant difference was analyzed by Student t test, where * *p* < 0.05, ** *p* < 0.01, compared to untreated cells.

To evaluate the efficiency of the CM derivative to inhibit cancer cell migration, scratch assay was performed on HepG2 and A549 cancer cell lines. As shown in Figure 3, the CM derivative significantly (*p* < 0.01) inhibited the percentage of wound closure to 24.05% ± 4.07 and 20.66% ± 3.84 in HepG2 and A549 cancer cells, respectively, compared to untreated cells. Meanwhile, etoposide, which was used as positive control, notably inhibited (*p* < 0.05) the percentage of wound closure to 32.44% ± 2.85 and 39.77% ± 3.41 in HepG2 and A549 cancer cells, respectively, compared to untreated cells.

### 2.4. Colony Formation Assay

**Figure 4:**
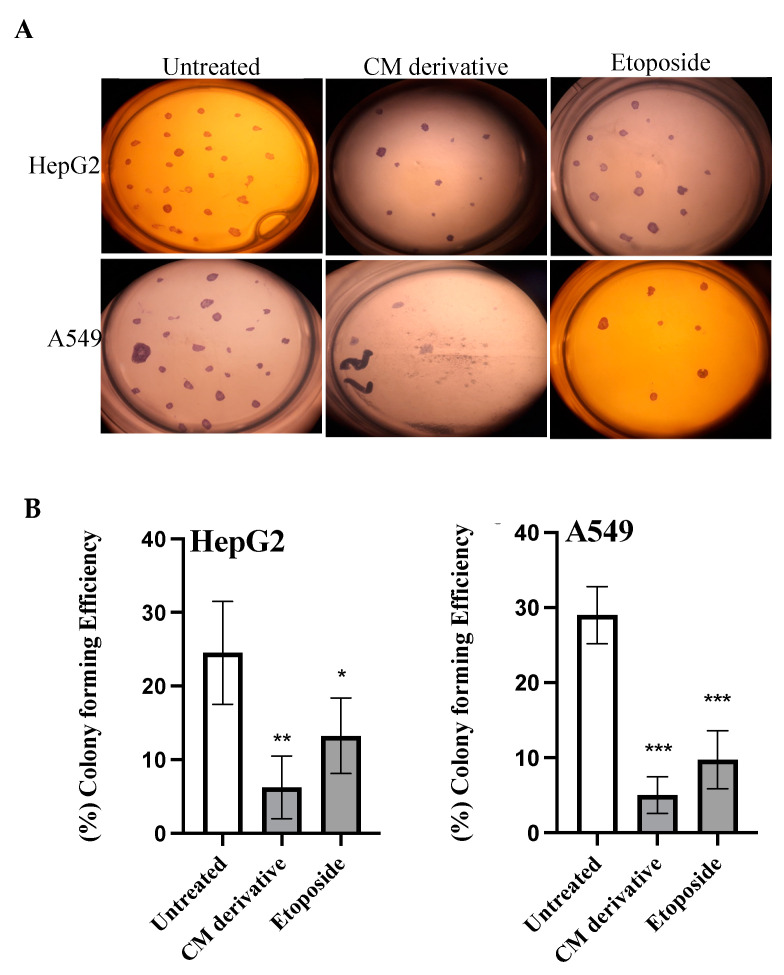
The effect of the CM derivative and etoposide on colony forming ability of HepG2 and A549 cancer cells. (**A**) Representative photos of colony formed with or without the treatment with the IC_50_ of CM derivative and etoposide. (**B**) The percentage of colony forming efficiency of HepG2 and A549 cancer cells. Bars represent mean ± SD. Significant difference was analysed using Student *t* test, where * *p* < 0.05, ** *p* < 0.01, *** *p* < 0.001, compared to untreated cells.

A colony formation assay was performed to investigate the ability of CM derivative to inhibit colonization of cancer cells. As shown in Figure 4, the CM derivative notably (*p* < 0.01) inhibited colony forming efficiency in HepG2 cancer cells to 6.25% ± 4.27, compared to untreated cells. Meanwhile, it significantly (*p* < 0.001) inhibited colonization in A549 cancer cells to 5.00% ± 2.44, compared to untreated cells. Meanwhile, etoposide, which was used as a positive control, showed a significant (*p* < 0.05, *p* < 0.01) inhibition of colonization in HepG2 and A549 cancer cells, respectively, compared to untreated cells.

### 2.5. Cell Cycle Analysis

**Figure 5:**
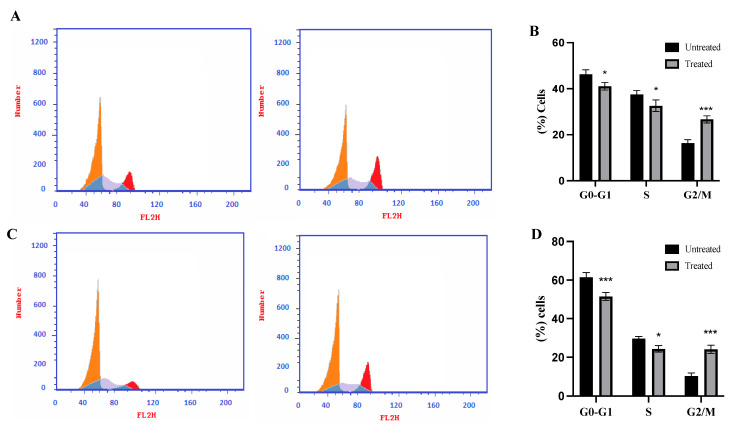
Effect of the CM derivative on cell cycle phase distribution of HepG2 and A549 cancer cell lines determined by flow cytometric analysis. (**A**) and (**C**) Representative histograms for HepG2 and A549 cancer cell lines, respectively, after PI staining. (**B**) and (**D**) Percentage of cells in the various cell cycle phases in HepG2 and A549 cancer cell lines, respectively. Data represent mean ± SD. Significant difference was analyzed by two-way ANOVA test, where: * *p* < 0.05, *** *p* < 0.001, compared to untreated cells.

To investigate cell cycle distribution before and after treatment with the CM derivative on HepG2 and A549 cancer cells, cell cycle analysis was performed using flowcytometry. As shown in Figure 5, G2/M phase showed significant (*p* < 0.001) increase in the percentage of cells to 26.68% ± 1.53 and 24.15% ± 2.08 in HepG2 and A549 cancer cells, respectively, when compared to untreated cells.

### 2.6. Annexin V Assay

**Figure 6:**
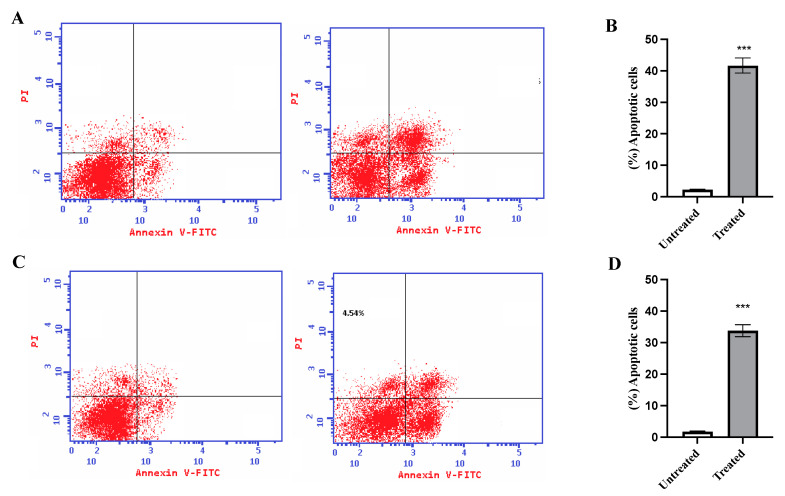
Effect of the CM derivative on apoptosis of HepG2 and A549 cancer cell lines estimated with Annexin V staining. (**A**) and (**C**) Representative dot plots for HepG2 and A549 cancer cell lines, respectively. (**B**) and (**D**) The percentage of total apoptotic cells in HepG2 and A549 breast cancer cell lines, respectively, after treatment with the IC_50_ of the CM derivative for 48 h. Data represent mean ± SD. Significant difference was analyzed by one-way ANOVA test, where: *** *p* < 0.0001, compared to untreated cells.

To investigate the ability of the CM derivative to induce apoptosis in HepG2 and A549 cancer cells, an Annexin V assay was performed. As shown in Figure 6, the total percentage of apoptosis increased significantly (*p* < 0.001) to 41.66% ± 2.403 and 33.78% ± 1.89 for HepG2 and A549 cancer cells, respectively.

### 2.7. Inhibition of Topo I/II Enzymes and Activation of Apoptotic Pathway

**Figure 7:**
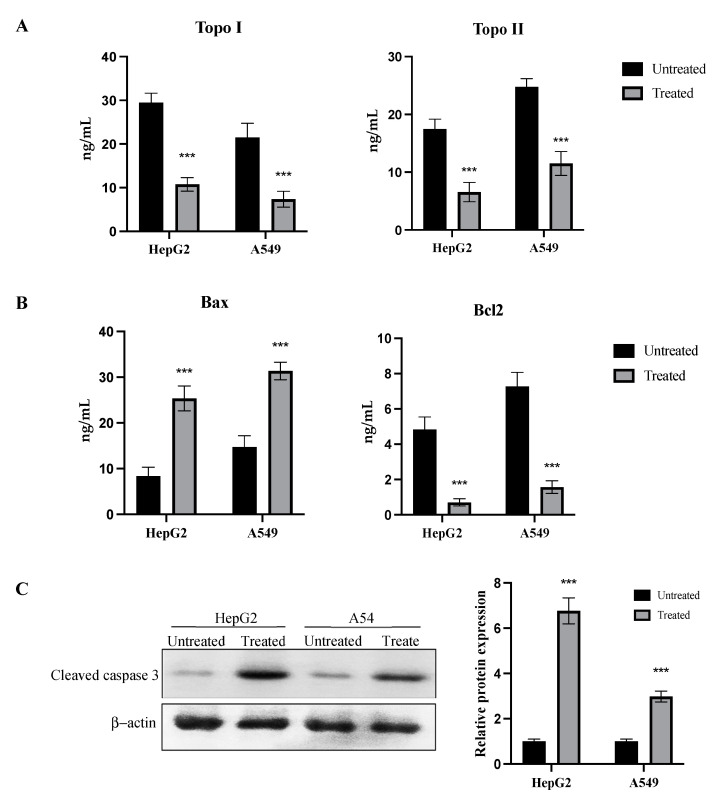
Dual inhibition of topo I/II enzyme activity and activation of apoptosis by the CM derivative. (**A**) Topo I and topo II enzyme activities in HepG2 and A549 cancer cells before and after treatment with the IC50 of the CM derivative. (**B**) Bax and bcl2 levels in HepG2 and A549 cancer cells before and after treatment with the IC50 of the CM derivative. (**C**) Expression of cleaved caspase 3 protein in HepG2 and A549 cancer cells treated with the IC 50 of the CM derivative estimated by western blotting. Expression was expressed relative to untreated cells after normalization to the corresponding β-actin protein expression. Bars represent mean ± SD. Significant difference was analyzed by one-way ANOVA test, where, *** *p* < 0.001, compared to untreated cells.

HepG2 and A549 cancer cells were treated with the IC50 of the CM derivative to evaluate the activity of topo I and topo II enzymes, and determine the levels of bax, and bcl2 proteins. As shown in Figure 7A, the CM derivative significantly (*p* < 0.001) inhibited the activity of topo I and topo II enzymes in HepG2 and A549 cancer cells, when compared to the untreated cells.

Bax levels were significantly (*p* < 0.001) increased after treatment with the CM derivative to 25.36 ng/mL ± 2.70 and 31.36 ng/mL ± 1.91 in HepG2 and A549 cancer cells, respectively. Meanwhile, bcl2 levels were significantly (*p* < 0.001) decreased to 0.7 ng/mL ± 0.2 and 1.57 ng/mL ± 0.35 in HepG2 and A549 cancer cells, respectively, as shown in Figure 7B.

To confirm the initiation of the apoptotic pathway, protein expression of cleaved caspase 3 was examined before and after treatment with the CM derivative in HepG2 and A549 cancer cells. As shown in Figure 7C, relative protein expression of cleaved caspase 3 was significantly (*p* < 0.001) increased to 6.77 ± 0.57 and 2.98 ± 0.24 in HepG2 and A549 cancer cells, respectively, when compared to the untreated cells.

### 2.8. LDH Release and Activation of Necro-Apoptotic Pathway

**Figure 8:**
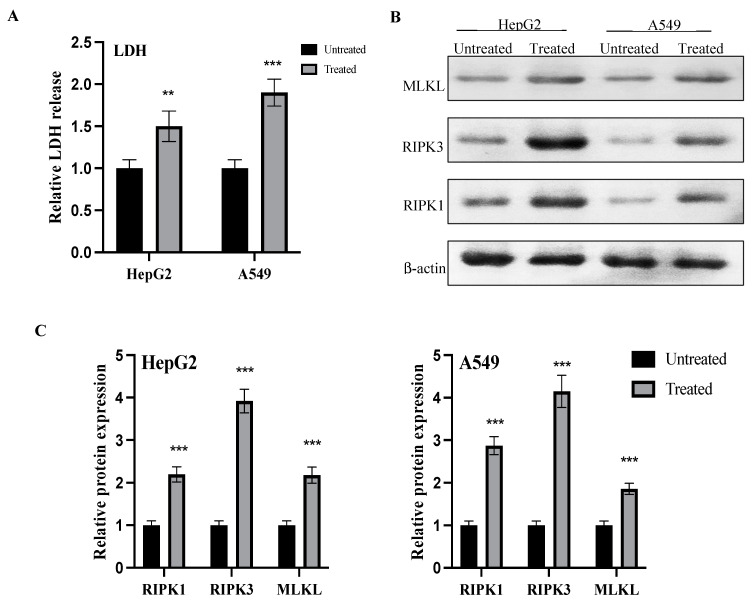
Effect of the CM derivative on LDH release and activation of the necro-apoptotic pathway. (**A**) Evaluation of LDH release before and after treatment with the IC50 of the CM derivative in HepG2 and A549 cancer cells. (**B**) Representative western blots of RIPK1, RIPK3, and MLKL proteins in HepG2 and A549 cancer cells treated with the IC 50 concentration of the CM derivative. β-actin was used as internal loading control. (**C**) Expression of proteins in HepG2 and A549 treated cells was expressed relative to untreated cells after normalization to the corresponding β-actin protein expression. Bars represent mean ± SD. Significant difference was analyzed by two-way ANOVA test, where, ** *p* < 0.01, *** *p* < 0.001, compared to untreated cells.

In the present study, lactate dehydrogenase (LDH) levels were evaluated to examine the initiation of necro-apoptotic pathway. After treatment with the IC50 of the CM derivative for 48 h in HepG2 and A549 cancer cells, LDH release was significantly (*p* < 0.01, *p* < 0.001) increased in HepG2 and A549 cancer cells, respectively, when compared to the untreated cells, as shown in Figure 8A.

Additionally, the expressions of receptor-interacting serine/threonine protein kinase 1 (RIPK1), receptor interacting serine/threonine protein kinase 3 (RIPK3), and mixed lineage kinase domain like (MLKL) proteins were evaluated before and after treatment of the IC50 of the CM derivative. As shown in Figure 8B,C, relative expressions of RIPK1, RIPK3, and MLKL proteins were significantly (0.001) elevated in both HepG2 and A549 cancer cells after treatment with the IC50 of the CM derivative, when compared to the untreated cells.

## 3. Discussion

Topoisomerase enzymes became a primary target in cancer therapy due to their crucial role in DNA supercoiling and entanglement [31]. Several studies reported the overexpression of topoisomerase in tumor cells. Therefore, they are considered a promising therapeutic approach in cancer treatment [32,33]. However, it was reported that targeting only one topo could trigger the over-release of the other and the simultaneous treatment with one topo inhibitor antagonizes its anticancer activity through the stimulation of the other topo enzyme [34]. Thus, the dual inhibition of topo I and topo II possess a substantial therapeutic advantage over single topo inhibitors.

**Figure 9:**
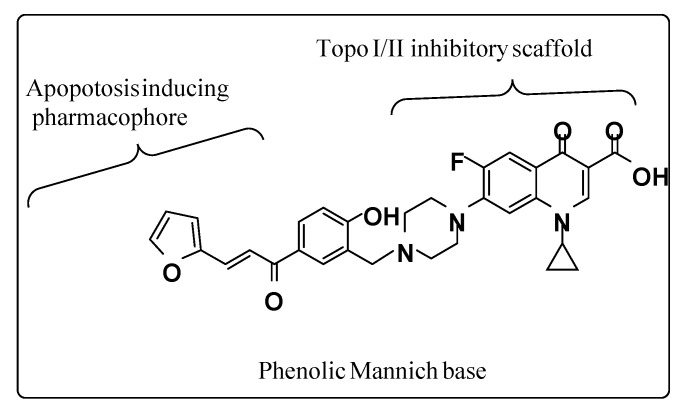
Illustrative structure of the CM derivative.

In the present study, the CM derivative is designed to combine two moieties with reported anti-proliferative activity in one of them through the Phenolic C-Mannich base (Figure 9). Such hybridization is supposed to achieve a synergistic effect on the anti-cancer activity of each one of them and enhance the potency of the newly formed compound [35,36].

The molecular docking results of the present study revealed that this CM derivative showed dual topo I and topo II binding and inhibition. It is noteworthy that the interactions profile shown by the phenolic chalcone Mannich derivative revealed two interactions with the enzyme one as H-donor with Asp479 amino acid residues and the other with Pro 819 amino acid as pi-H and two hydrophobic interactions with DNA nucleobases (DA 12, DT 9) as H-pi between piperazine moiety and the two adjacent nucleobases.

Our molecular docking findings paved the way for further investigations on the anticancer activity of this CM derivative. At first, the CM derivative was screened against different cancer cell lines, including MCF-7, HepG2, A549, and M14, in addition to the normal cell line, Wi38, to evaluate its cytotoxic activity. Our findings revealed that the CM derivative showed more potent antiproliferative activity against both HepG2 and A549 cancer cell lines and weak antiproliferative activity against MCF-7 and M14 cancer cell lines. Therefore, HepG2 and A549 cancer cells were selected for further investigations on the CM derivative in the present study. Furthermore, it is worth mentioning that the cytotoxic activity of the CM derivative against Wi38 normal cells was observed at high concentrations, while its potent antiproliferative activity against HepG2 and A549 cancer cells was obtained at lower concentrations.

Further investigations on the activity of the CM derivative against HepG2 and A549 cancer cells were carried out in the present study through examining its effect on cancer cells to migrate and form colonies. Our CM derivative suppressed the migration of cancer cells and the ability of forming colonies in both HepG2 and A549 cancer cell lines. This is critical for presenting that the anticancer activity of this CM derivative extends to inhibit distant invasion and colonization of tumor cells, which is a remarkable and essential feature for any new anticancer candidate [37].

Additionally, Topoisomerase inhibitors are central biological targets that initiate many signaling pathways, including apoptotic and necro-apoptotic pathways [31,38]. Both apoptosis and necro-apoptosis pathways are involved in the progression of various types of cancer, comprising of hepatocellular carcinoma, breast cancer, colorectal cancer, and head and neck squamous cell carcinoma [10,39,40].

Regarding apoptotic pathways, inhibition of topoisomerases leads to DNA damage and induction of programmed death through the activation of caspases and pro-apoptotic factors [38,41]. In the present study, our findings revealed that the CM derivative showed dual notable inhibition of topo I and topo II, which resulted in the initiation of the intrinsic apoptotic pathway and the activation of caspases cascade. In addition, bax levels were elevated and the bcl2 levels were suppressed following the treatment with the CM derivative which led to a notable increase in the protein expression of caspase 3 and induction of apoptotic signaling pathway. This is aligned with the notable increase in the total percentage of apoptotic cells and the arrest of the cell cycle at G2/M phase, which were exerted by our CM derivative.

**Figure 10:**
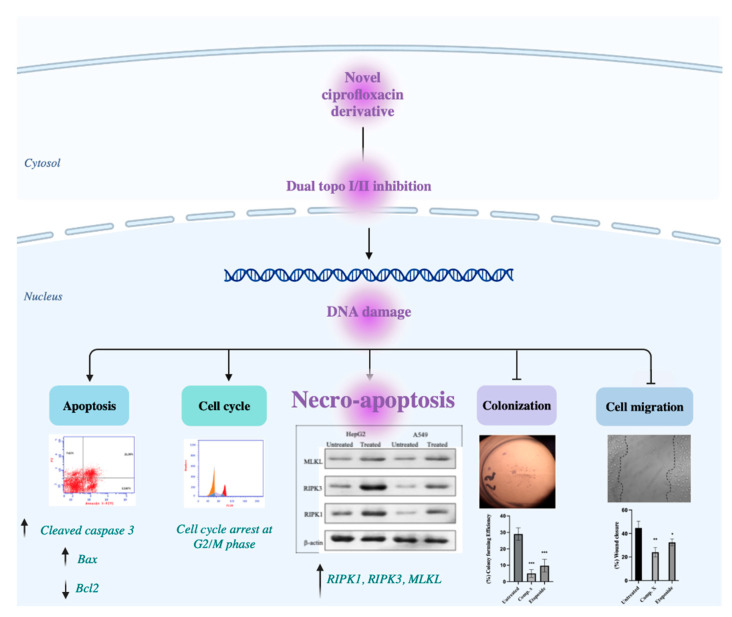
Illustrative figure summarizes the molecular mechanism of the anti-proliferative, anti-migration, apoptotic and necro-apoptotic activities of the CM derivative against cancer cells.

However, one of the major obstacles in the field of cancer therapy is the multi-drug resistance, which leads to the escape of the programmed cell death. The necro-apoptotic pathway holds an advantage to initiate another form of cell death to tumors that show resistance to the apoptotic machinery pathways. Necro-apoptosis is a programmed lytic cell death, which is considered a promising novel target for cancer therapy. It was documented that inhibition of topoisomerases also leads to the activation of the necro-apoptotic pathway [42,43]. The necro-apoptotic pathway is driven by a signaling cascade of RIPK1/RIPK3/MLKL [44]. RIPK1 is a key regulator in cell survival and cell death that controls the cell’s fate in response to cellular stress. RIPK1 activates the necro-apoptotic pathway with further induction of RIPK3, followed by MLKL over-expression [45]. Furthermore, it was reported that the loss of RIPK3 expression is a feature for many tumors [46]. Many studies have developed small molecules targeting necro-apoptosis for cancer therapy [30,47]. Our findings revealed that the dual topo I and topo II inhibition that was exerted by our CM derivative resulted in the activation of RIPK1/RIPK3/MLKL pathway, which led to the activation of necro-apoptotic pathway and cell death, as shown in Figure 10. The inability to induce apoptosis may result in mutations, leading to carcinogenesis. However, the cell death, indicated by the release of LDH which is induced by necro-apoptosis, is an important aspect for looking for therapeutic candidates to treat cancer [48,49]. The LDH release after treatment with the CM derivative came to support our hypothesis that our CM derivative initiated and activated the necro-apoptotic pathway.

## 4. Materials and Methods

### 4.1. Synthesis of (E)-1-cyclopropyl-6-fluoro-7-(4-(5-(3-(furan-2-yl) acryloyl)-2 hydroxybenzyl) piperazin-1-yl)-4-oxo-1,4-dihydroquinoline-3-carboxylic acid

#### 4.1.1. Preparation of the Chalcone

To a mixture of furfural (6.10 mmol) and 4-hydroxy acetophenone (5.55 mmol) in ethanol (15 mL), we slowly added an aqueous solution of KOH (50%, 62 mL), and stirred vigorously under an argon atmosphere at room temperature for 12–24 h. Ethanol was removed under reduced pressure. The residue was diluted in ice water and acidified with an aqueous solution of hydrochloric acid (10%), filtered, and washed with water. The crude product was recrystallized with ethanol to afford the derivative [50].

#### 4.1.2. Preparation of the Mannich Base

**Scheme 1:**
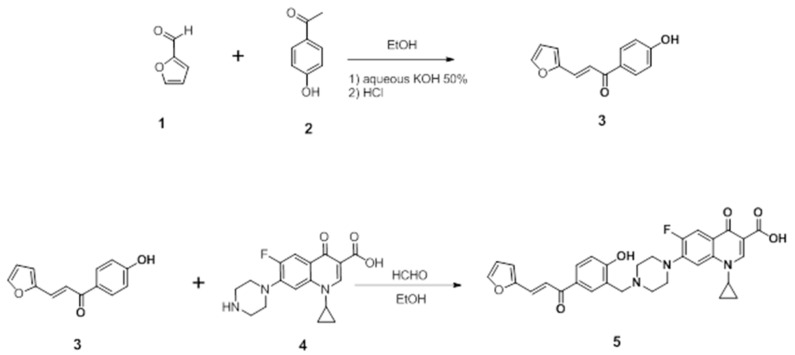
Synthesis of (*E*)-1-cyclopropyl-6-fluoro-7-(4-(5-(3-(furan-2-yl) acryloyl)-2 hydroxybenzyl) piperazin-1-yl)-4-oxo-1,4-dihydroquinoline-3-carboxylic acid.

To an equimolar mixture of ciprofloxacin and chalcone (3) in ethanol, 1 mL of formaldehyde (37%) was added. The reaction mixture was heated at reflux for (36) hours and cooled. The precipitated solid was filtered off under vacuum. Crystallization from aqueous ethanol afforded the Mannich bases 5 in 58% yield, as shown in Scheme 1. The CM derivative was identified by ^1^H-NMR and ^13^C-NMR spectral data, shown in the Supplementary Materials.

### 4.2. Docking Studies of the CM Derivative on Human Topo I and Topo II Enzymes

The molecular docking study for the CM derivative was carried out using “Molecular Operating Environment 2019.0102 software (MOE)”. The X-ray crystallographic structure of protein targets of topoisomerase I and topoisomerase IIB were obtained from the Protein Data Bank through the internet (http://www.rcsb.org/pdb/, accessed on 5 February 2022, code and 1T8I and 3QX3). Topo I and topo II B were complexed with their ligands camptothecin and etoposide, respectively.

To validate our study, the ligand was re-docked with the active pocket site of the topo I enzyme. It showed interactions with the receptor by Hb formations with Arg 364, Asp 533 and Lys 532 residues and hydrophobic interactions as arene–arene interactions or pi–pi stacking with DNA nucleotide bases (DC 112, TGP 11 and DA113) for topo I. The docking algorithm was able to predict the co-crystalized ligand pose with least RMSD with an energy score of −9.535 kcal/mol.

### 4.3. Cell Culture

MCF-7, HepG2, A549, M14, and Wi38 cell lines were obtained from American type culture collection (ATCC, Manassas, VA, USA). Fresh Dulbecco’s Modified Eagle’s Medium (DMEM, Sigma-Aldrich, Inc., St Louis, MO, USA) was used as a culture medium. The 10% fetal bovine serum (FBS, Biosolutions International, Melbourne, Australia), 1% penicillin-streptomycin mixture (Invitrogen, Grand Island, NY, USA), and 1% L-glutamine (Sigma-Aldrich, Inc.) were added to the culture medium in a humidified 5% CO_2_ atmosphere at 37 °C.

### 4.4. Cell Viability

Cell viability assay was achieved using MTT reagent [3-(4, 5-dimethyl thiazol-2yl)-2, 5-diphenyltetrazolium bromide]. A total of 10^4^ cells of MCF-7, HepG2, A549, M14, and Wi38 cells were seeded per well in triplicate in 96-well plates and allowed to grow in fresh DMEM medium for 24 hours. Then, medium was changed with fresh DMEM containing different concentrations (31.25, 62.5, 125, 250, 500, 1000 μg/mL) of the CM derivative. After incubation for 48 hours, 10 μL of MTT (5 μg/mL) was added per well and incubated in the dark for 3 hours at 37 °C. A total of 100 μL of DMSO was added to dissolve the Formazan crystals that were formed. Absorbance was measured using an ELISA reader at 570 nM [51]. The IC_50_ of the compound was calculated for each cell line using Graph Pad Prism-9 software.

Further investigations on the efficacy and activity of the CM derivative were continued in the present study on cancer cells that showed lower IC_50_.

Additionally, etoposide (#E1383, Sigma-Aldrich, St Louis, MO, USA) was used as a positive control in the present study at concentrations of 31.25, 62.5, 125, 250, 500, and 1000 μg/mL.

### 4.5. Cell Migration Assay

Cells (1×10^6^ cells) were seeded in 6-well plate culture dish in DMEM medium and incubated at 37 °C in humidified 5% CO_2_ conditions until confluent. Then, using 200 µl tips, a scratch was created in the formed monolayers. An angle of around 30 degrees was utilized to keep the scratch width limited. PBS was used to wash the cells followed by treatment with the IC_50_ of the CM derivative or etoposide in DMEM and incubation for 48 h. Using a live-cell imaging system, photos were captured using the cytosmart system. The estimation of the open scratch area was performed at different points utilizing ImageJ, covering all the distances that cells had migrated, and data were analysed using GraphPad Prism-9 software.

### 4.6. Colony Formation Assay

In triplicate, 100 cells were seeded per well in 24-well plates and allowed to attach for 24 h in fresh DMEM medium. Then, cells were incubated for 48 h in DMEM containing IC_50_ of the CM derivative or etoposide. PBS was used to wash the cells twice. Then, the cells were allowed to grow in fresh DMEM medium for 14 days at 37 °C under humidified 5% CO_2_ conditions, and the medium was changed on a regular basis. Then, 4% formaldehyde was used to fix the cells followed by washing with PBS and staining for 15 min with crystal violet. Manual counting using stereo microscope (Jenco^™^ stereo microscopes, GL series, Sigma Aldrich Co.) was used to estimate the total number of colonies. Colony forming efficiency (CFE) was assessed by the following equation:

$$\mathrm{CFE}=\frac{\mathrm{numberof}\;\mathrm{colonies}\;\mathrm{formed}}{\mathrm{number}\;\mathrm{of}\;\mathrm{plated}\;\mathrm{cells}}\times 100\%$$

### 4.7. Cell cycle Analysis

Propidium iodide staining was used in flowcytometric analysis to evaluate cell cycle analysis. The experiment was done in triplicate. A total of 1×10^6^ cells of both untreated and treated cancer cells with the IC_50_ of the CM derivative for 48 h were collected by centrifugation, washed with PBS (Sigma-Aldrich, Inc.) and fixed with ice cold 66% ethanol. The fixed cells were washed with PBS and resuspended for 30 min in 1 mg/mL RNAse. Propidium iodide (50 μg/mL) was used to label the intracellular DNA by incubating the cells for at least 20 min at 4 °C in dark. Then, samples were analysed using flow cytometry.

### 4.8. Annexin V assay

An Annexin V-FITC Apoptosis Detection Kit (Sigma-Aldrich) was used to detect apoptosis using flowcytometric analysis. Using the IC_50_ of the CM derivative, the cancer cells were treated for 48 h, followed staining the cells according to the manufacturer’s instructions. Then, cell fluorescence was determined immediately with a flow cytometer (Becton Dickinson, Franklin Lakes, NJ, USA), counting at least 10^4^ cells. Dot plots were obtained, and % of apoptosis was estimated.

### 4.9. Evaluation the Activity on Topo I, Topo II, Bax, and bcl2

To evaluate the inhibitory activity of the CM derivative against topo I and topo II enzymes, human DNA topoisomerase 1 ELISA kit (#MBS289119, MyBioSource, San Diego, CA, USA) and human DNA topoisomerase II ELISA kit (#EKN48879-96T, BIOMATIK, Wilmington, DE, USA) were utilized. Also, to evaluate the level of bax (proapoptotic factor) and bcl2 (antiapoptotic factor) proteins, (#E-EL-H0562, human bax ELISA kit, Elabscience, TX, USA) and (#E-EL-H0114, human bcl2 ELISA kit, Elabscience, Houston, TX, USA) were utilized, respectively. Cancer cells were treated with the IC50 of the CM derivative for 48 h. Then, untreated and treated cells were harvested followed by measurement of topo I, topo II, bax, and bcl2 according to manufacturer’s instructions. A standard curve was obtained, and the absorbance was measured at 450 nm. The concentration of topo I, topo II, bax, and bcl2 were calculated from the standard curve. The experiment was performed in triplicate.

### 4.10. Lactate Dehydrogenase Release Assay

To evaluate the cytotoxic activity of the CM derivative against HepG2 and A549 cancer cells, an LDH release assay (#ab65393, LDH cytotoxicity assay kit, abcam, Cambridge, UK) was utilized. Cancer cells were treated with the IC50 of the CM derivative for 48 h, then LDH release was determined according to manufacturer’s instructions. Cell cultures were centrifuged for 10 min. A total of 10 μL of the supernatant was placed into 96-well plate. Then, 100 μL of LDH Rx buffer was added to each well followed by an incubation for 30 min. The absorbance was measured at 490 nm.

### 4.11. Analysis of Protein Expression via Western Blotting

To examine the expression levels of RIPK1, RIPK3, MLKL, and cleaved caspase 3 proteins, sodium dodecyl sulphate–polyacrylamide gel electrophoresis (SDS-PAGE) analysis was accomplished. Cells were treated with the IC_50_ of the CM derivative for 48 h. Then, total protein was extracted in RIPA lysis buffer, containing 50 mM Tris–Cl, pH 7.5; 0.1% SDS, 150 mM NaCl, 0.5% sodium deoxycholate, 1 mM PMSF, and 1% Nonidet P-40, supplemented with the complete protease inhibitor cocktail (Roche, Mannheim, Germany). To determine total protein concentration, the Bradford method was used [52]. SDS-PAGE (15% acrylamide) was used to separate cell lysates containing 30 μg protein. Then, they were transferred to a Hybond™ nylon membrane (GE Healthcare) followed by an incubation in Blocking Solution for 1 hour at room temperature. Membranes were incubated at 4 °C overnight with RIPK1, RIPK3, MLKL, and cleaved caspase 3 antibodies (New England Biolabs, Ipswich, MA, USA) diluted (1:1000) with PBS. Then, 30–60 min washing of membranes was performed followed by an incubation at room temperature for 1 h with the HRP-conjugated secondary antibody (New England Biolabs) diluted (1:1000) in PBS [53]. Immunoreactive proteins were detected using an enhanced chemiluminescence kit (GE Healthcare, Little Chalfont, UK) by a luminescent image analyser (LAS-4000, Fujifilm Co., Tokyo, Japan), according to the manufacturer’s instructions. A loading control was utilised, an antibody against β-actin (New England Biolabs) (1:1000), to detect β-actin. Electrophoresis and electroblotting, using a discontinuous buffer system, were carried out in a Bio-Rad Trans-Blot SD Cell apparatus (Bio-Rad, Hercules, CA, USA). Densitometric analysis was then performed by using The Image Processing and Analysis Java (ImageJ) program. Data were normalized to β-actin levels.

### 4.12. Statistical Analysis

At least three independent experiments were used to obtain the results. Data were expressed as mean ± standard deviation. Student’s t-test was used to analyse differences after one- or two-way analysis of variance (ANOVA), with the use of GraphPad Prism 9 statistical software (GraphPad, La Jolla, CA, USA) and Excel software (Microsoft, Redwood, WA, USA). Differences were considered significant when the probability values (*p*) were less than 0.05.

## 5. Conclusions

The present study synthesized a novel C7-piperazinyl ciprofloxacin *ortho*-phenolic chalcone Mannich base derivative, CM derivative, that exerted substantial dual inhibition of topo I/II enzyme activities. Also, it showed antiproliferative activity, suppressed the cell migration and colony formation abilities for A549 and HepG2 cancer cell lines and activated the apoptotic pathway through the caspase 3 signaling cascade. In addition, the CM derivative initiated another backup deadly pathway, necro-apoptosis, through the activation of the RIPK1/RIPK3/MLKL pathway. However, further investigations against different cancer cell lines are required for more understanding of its efficacy, safety, and mechanism of action.

## Figures and Tables

**Table 1:** Receptor interactions and binding energies of the CM derivative and camptothecin at the active site of Topo I enzyme (PDB code:1T8I).

NO	Compound	S ^a^ kcal/mole	RMSD_Refine ^b^	Amino Acid Bond	Distance Å	E (Kcal mol)
1	CM derivative	−11.605	1.901	Asp 533/H-donor	3.34	−0.7
				Lys 532/H-acceptor	3.67	−0.9
				TGP 11/pi-pi	3.95	0.0
				DA 113/H-pi	3.52	−0.6
2	Camptothecin	−9.9577	1.390	Asp 533/H-donor	3.06	−2.9
				Lys 532/H-acceptor	3.10	−3.0
				Arg 364/H-acceptor	3.57	−1.6
				TGP 11/pi-pi	3.88	0.0
				DA 113/pi-pi	3.56	0.0
				DC 112/pi-pi	3.68	0.0

^a^ S: the score of a compound placement inside the protein binding pocket. ^b^ RMSD_Refine: the root-mean-squared-deviation (RMSD) between the predicted pose and those of the crystal one (after and before refinement process, respectively).

**Table 2:** Receptor interactions and binding energies of the CM derivative and etoposide at the active site of the Topo II enzyme (PDB code:3QX3).

NO	Compound	S ^a^ kcal/mole	RMSD_Refine ^b^	Amino acid bond	Distance Å	E (Kcal mol)
1	CM derivative	−8.247	1.727	Gln789/H-donor	2.79	−7.5
				Pro 819/pi-H	4.12	−0.5
				DA 12/H-pi	4.01	−0.7
				DT 9/H-pi	4.33	−0.5
2	Etoposide	−7.671	1.603	Asp 479/H-donor	3.08	−2.3
				DG 13/H-donor	3.06	−2.0
				DA 12/H-pi	3.57	−0.6

^a^ S: the score of a compound placement inside the protein binding pocket. ^b^ RMSD_Refine: the root-mean-squared-deviation (RMSD) between the predicted pose and those of the crystal one (after and before refinement process, respectively).

**Table 3:** IC50 concentrations of CM derivative and etoposide against different cell lines.

Cell Lines	IC50 (Mean ± SD) (μg/mL)	
	CM Derivative	Etoposide
MCF-7	51.58 ± 4.82	ND
HepG2	22.09 ± 1.7	34.32 ± 2.81
A549	27.71 ± 2.4	48.32 ± 3.93
M14	90.48 ± 7.4	ND
Wi38	118.65 ± 10.3	ND

ND; not determined.

## Data Availability

All data are fully available and included in the manuscript.

## Supplementary Materials

The following supporting information can be downloaded at: https://www.mdpi.com/article/10.3390/molecules27227993/s1, Figure S1: ^1^H-NMR spectrum of compound **5** (400 MHz, *DMSO-d6*); Figure S2: ^13^C-NMR spectrum of compound **5** (400 MHz, *DMSO-d6*).

## Sample Availability

Samples of the compound (the CM derivative) are available from the authors.

## References

[1] Gouzerh F., Bessière J.-M., Ujvari B., Thomas F., Dujon A.M., Dormont L. (2022). Odors and cancer: Current status and future directions. Biochim. Et Biophys. Acta (BBA)—Rev. Cancer.

[2] Abdel-Hamid N.M., Fathy M., Koike C., Yoshida T., Okabe M., Zho K., Abouzied M., Nikaido T. (2021). Identification of Chemo and Radio-Resistant Sub-Population of Stem Cells in Human Cervical Cancer HeLa Cells. Cancer Investig..

[3] Noto Z., Yoshida T., Okabe M., Koike C., Fathy M., Tsuno H., Tomihara K., Arai N., Noguchi M., Nikaido T. (2013). CD44 and SSEA-4 positive cells in an oral cancer cell line HSC-4 possess cancer stem-like cell characteristics. Oral Oncol..

[4] Wang F., Yoshida T., Okabe M., Fathy M., Sun Y., Koike C., Salto S., Nikaido T. (2017). CD24+SSEA4+cells in Ovarian Carcinoma Cells Demonstrated the Characteristics as Cancer Stem Cells. J. Cancer Sci. Ther..

[5] Abdellatef A.A., Fathy M., Mohammed A.E.I., Bakr M.S.A., Ahmed A.H., Abbass H.S., El-Desoky A.H., Morita H., Nikaido T., Hayakawa Y. (2021). Inhibition of cell-intrinsic NF-kappaB activity and metastatic abilities of breast cancer by aloe-emodin and emodic-acid isolated from Asphodelus microcarpus. J. Nat. Med..

[6] Fathy M., Nikaido T. (2013). In vivo modulation of iNOS pathway in hepatocellular carcinoma by Nigella sativa. Env. Health Prev. Med..

[7] Fathy M., Nikaido T. (2018). In vivo attenuation of angiogenesis in hepatocellular carcinoma by Nigella sativa. Turk. J. Med. Sci..

[8] Liang X., Wu Q., Luan S., Yin Z., He C., Yin L., Zou Y., Yuan Z., Li L., Song X. (2019). A comprehensive review of topoisomerase inhibitors as anticancer agents in the past decade. Eur. J. Med. Chem..

[9] Khadka D.B., Cho W.-J. (2013). Topoisomerase inhibitors as anticancer agents: A patent update. Expert Opin. Ther. Pat..

[10] Lin C., Chang T., Hsieh W., Hung M., Lin I., Lai S., Tzeng Y. (2016). Simultaneous induction of apoptosis and necroptosis by Tanshinone IIA in human hepatocellular carcinoma HepG2 cells. Cell Death Discov..

[11] Hooper D.C. (1999). Mode of Action of Fluoroquinolones. Drugs.

[12] Alaaeldin R., Mustafa M., Abuo-Rahma G.E.-D.A., Fathy M. (2022). In vitro inhibition and molecular docking of a new ciprofloxacin-chalcone against SARS-CoV-2 main protease. Fundam. Clin. Pharmacol..

[13] Alaaeldin R., Abuo-Rahma G.E.-D.A., Zhao Q.-L., Fathy M. (2021). Modulation of apoptosis and epithelial-Mesenchymal transition E-cadherin/TGF-β/Snail/TWIST pathways by a new ciprofloxacin chalcone in breast cancer cells. Anticancer Res..

[14] Esfandiari Mazandaran K., Mirshokraee S.A., Didehban K., Houshdar Tehrani M.H. (2019). Design, Synthesis and Biological Evaluation of Ciprofloxacin- Peptide Conjugates as Anticancer Agents. Iran. J. Pharm. Res..

[15] Zandi A., Moini Zanjani T., Ziai S.A., Khazaei Poul Y., Haji Molla Hoseini M. (2017). Evaluation of the cytotoxic effects of ciprofloxacin on human glioblastoma A-172 cell line. Middle East. J. Cancer.

[16] Chrzanowska A., Roszkowski P., Bielenica A., Olejarz W., Stępień K., Struga M. (2020). Anticancer and antimicrobial effects of novel ciprofloxacin fatty acids conjugates. Eur. J. Med. Chem..

[17] Kloskowski T., Gurtowska N., Nowak M., Joachimiak R., Bajek A., Olkowska J., Drewa T. (2011). The influence of ciprofloxacin on viability of A549, HepG2, A375. S2, B16 and C6 cell lines in vitro. Acta Pol. Pharm..

[18] Eisa M.A., Fathy M., Abuo-Rahma G., Abdel-Aziz M., Nazmy M.H. (2021). Anti-Proliferative and Pro-Apoptotic Activities of Synthesized 3,4,5 Tri-Methoxy Ciprofloxacin Chalcone Hybrid, through p53 Up-Regulation in HepG2 and MCF7 Cell Lines. Asian Pac. J. Cancer Prev..

[19] Eisa M.A., Fathy M., Nazmy M.H. (2021). Potential COX2 mediated therapeutic effect of ciprofloxacin. Minia J. Med. Res..

[20] Alaaeldin R., Nazmy M.H., Abdel-Aziz M., Abuo-Rahma G.E.-D.A., Fathy M. (2020). Cell Cycle Arrest and Apoptotic Effect of 7-(4-(N-substituted carbamoylmethyl) piperazin-1-yl) Ciprofloxacin-derivative on HCT 116 and A549 Cancer Cells. Anticancer Res..

[21] Abd-Elhamid R.A., Nazmy M.H., Fathy M. (2020). Targeting Apoptosis as a Therapeutic Approach in Cancer. Minia J. Med. Res..

[22] Abdel-Aal M.A., Abdel-Aziz S.A., Shaykoon M.S.A., Abuo-Rahma G.E.D.A. (2019). Towards anticancer fluoroquinolones: A review article. Arch. Der Pharm..

[23] Fathy M., Sun S., Zhao Q.-L., Abdel-Aziz M., Abuo-Rahma G.E.-D.A., Awale S., Nikaido T. (2020). A New Ciprofloxacin-derivative Inhibits Proliferation and Suppresses the Migration Ability of HeLa Cells. Anticancer Res..

[24] Fathy M., Khalifa E., Fawzy M.A. (2019). Modulation of inducible nitric oxide synthase pathway by eugenol and telmisartan in carbon tetrachloride-induced liver injury in rats. Life Sci..

[25] Fawzy M.A., Maher S.A., Bakkar S.M., El-Rehany M.A., Fathy M. (2021). Pantoprazole Attenuates MAPK (ERK1/2, JNK, p38)-NF-kappaB and Apoptosis Signaling Pathways after Renal Ischemia/Reperfusion Injury in Rats. Int. J. Mol. Sci.

[26] Fawzy M.A., Maher S.A., El-Rehany M.A., Welson N.N., Albezrah N.K.A., Batiha G.E.-S., Fathy M. (2022). Vincamine Modulates the Effect of Pantoprazole in Renal Ischemia/Reperfusion Injury by Attenuating MAPK and Apoptosis Signaling Pathways. Molecules.

[27] Oba J., Okabe M., Yoshida T., Soko C., Fathy M., Amano K., Kobashi D., Wakasugi M., Okudera H. (2020). Hyperdry human amniotic membrane application as a wound dressing for a full-thickness skin excision after a third-degree burn injury. Burn. Trauma.

[28] Okabe M., Yoshida T., Suzuki M., Goto M., Omori M., Taguchi M., Toda A., Suzuki T., Nakagawa K., Hiramoto F. (2017). Hyperdry Human Amniotic Membrane (HD-AM) is Supporting Aciclovir Included Device of Poly-N-p-Vinylbenzyl-D-Lactonamide (PVLA) Sphere for Treatment of HSV-1 Infected Rabbit Keratitis Model. J. Biotechnol. Biomater..

[29] Shytaj I.L., Fares M., Gallucci L., Lucic B., Tolba M.M., Zimmermann L., Adler J.M., Xing N., Bushe J., Gruber A.D. (2022). The FDA-Approved Drug Cobicistat Synergizes with Remdesivir To Inhibit SARS-CoV-2 Replication In Vitro and Decreases Viral Titers and Disease Progression in Syrian Hamsters. mBio.

[30] Zang X., Song J., Li Y., Han Y. (2022). Targeting necroptosis as an alternative strategy in tumor treatment: From drugs to nanoparticles. J. Control. Release.

[31] Xiong K., Qian C., Yuan Y., Wei L., Liao X., He L., Rees T.W., Chen Y., Wan J., Ji L. (2020). Necroptosis induced by ruthenium (II) complexes as dual catalytic inhibitors of topoisomerase I/II. Angew. Chem. Int. Ed..

[32] Hevener K., Verstak T.A., Lutat K.E., Riggsbee D.L., Mooney J.W. (2018). Recent developments in topoisomerase-targeted cancer chemotherapy. Acta Pharm. Sin. B.

[33] Thakur D.S. (2010). Topoisomerase II inhibitors in cancer treatment. Int. J. Pharm. Sci. Nanotechnol..

[34] Rasheed Z.A., Rubin E.H. (2003). Mechanisms of resistance to topoisomerase I-targeting drugs. Oncogene.

[35] Tugrak M., Yamali C., Sakagami H., Gul H.I. (2016). Synthesis of mono Mannich bases of 2-(4-hydroxybenzylidene)-2, 3-dihydroinden-1-one and evaluation of their cytotoxicities. J. Enzym. Inhib. Med. Chem..

[36] Canturk P., Kucukoglu K., Topcu Z., Gul M., Gul H.I. (2008). Effect of some bis Mannich bases and corresponding piperidinols on DNA topoisomerase I. Arzneim. -Forsch..

[37] Sabra R.T., Abdellatef A.A., Abdel-Sattar E., Fathy M., Meselhy M.R., Hayakawa Y. (2022). Russelioside A, a Pregnane Glycoside from *Caralluma tuberculate*, Inhibits Cell-Intrinsic NF-κB Activity and Metastatic Ability of Breast Cancer Cells. Biol. Pharm. Bull..

[38] Sordet O., Khan Q.A., Kohn K.W., Pommier Y. (2003). Apoptosis induced by topoisomerase inhibitors. Curr. Med. Chem.-Anti-Cancer Agents.

[39] Zhao W., Feng H., Sun W., Liu K., Lu J.-J., Chen X. (2017). Tert-butyl hydroperoxide (t-BHP) induced apoptosis and necroptosis in endothelial cells: Roles of NOX4 and mitochondrion. Redox Biol..

[40] Zhu Y., Cui H., Xia Y., Gan H. (2016). RIPK3-mediated necroptosis and apoptosis contributes to renal tubular cell progressive loss and chronic kidney disease progression in rats. PLoS ONE.

[41] Alaaeldin R., Hassan H.A., Abdel-Rahman I.M., Mohyeldin R.H., Youssef N., Allam A.E., Abdelwahab S.F., Zhao Q.-L., Fathy M. (2022). A New EGFR Inhibitor from Ficus benghalensis Exerted Potential Anti-Inflammatory Activity via Akt/PI3K Pathway Inhibition. Curr. Issues Mol. Biol..

[42] Liu X., Xie X., Ren Y., Shao Z., Zhang N., Li L., Ding X., Zhang L. (2021). The role of necroptosis in disease and treatment. MedComm.

[43] Alaaeldin R., Abdel-Rahman I.A.M., Hassan H.A., Youssef N., Allam A.E., Abdelwahab S.F., Zhao Q.-L., Fathy M. (2021). Carpachromene Ameliorates Insulin Resistance in HepG2 Cells via Modulating IR/IRS1/PI3k/Akt/GSK3/FoxO1 Pathway. Molecules.

[44] Saeed W.K., Jun D.W. (2014). Necroptosis: An emerging type of cell death in liver diseases. World J. Gastroenterol..

[45] Lu Z., Wang H., Zhu M., Song W., Wang J., Wu C., Kong Y., Guo J., Li N., Liu J. (2018). Ophiopogonin D′, a natural product from radix ophiopogonis, induces in vitro and in vivo RIPK1-dependent and caspase-independent apoptotic death in androgen-independent human prostate cancer cells. Front. Pharmacol..

[46] Najafov A., Chen H., Yuan J. (2017). Necroptosis and Cancer. Trends Cancer.

[47] Su Z., Yang Z., Xie L., DeWitt J., Chen Y. (2016). Cancer therapy in the necroptosis era. Cell Death Differ..

[48] Fathy M., Fawzy M.A., Hintzsche H., Nikaido T., Dandekar T., Othman E.M. (2019). Eugenol exerts apoptotic effect and modulates the sensitivity of HeLa cells to cisplatin and radiation. Molecules.

[49] Chou H.L., Fong Y., Wei C.K., Tsai E.M., Chen J.Y., Chang W.T., Wu C.Y., Huang H.W., Chiu C.C. (2017). A Quinone-Containing Compound Enhances Camptothecin-Induced Apoptosis of Lung Cancer Through Modulating Endogenous ROS and ERK Signaling. Arch. Immunol. Exp..

[50] Karki R., Song C., Kadayat T.M., Magar T.B., Bist G., Shrestha A., Na Y., Kwon Y., Lee E.S. (2015). Topoisomerase I and II inhibitory activity, cytotoxicity, and structure-activity relationship study of dihydroxylated 2,6-diphenyl-4-aryl pyridines. Bioorg. Med. Chem..

[51] Goel A., Prasad A.K., Parmar V.S., Ghosh B., Saini N. (2009). Apoptogenic effect of 7,8-diacetoxy-4-methylcoumarin and 7,8-diacetoxy-4-methylthiocoumarin in human lung adenocarcinoma cell line: Role of NF-kappaB, Akt, ROS and MAP kinase pathway. Chem. Biol. Interact..

[52] Bradford M.M. (1976). A rapid and sensitive method for the quantitation of microgram quantities of protein utilizing the principle of protein-dye binding. Anal. Biochem..

[53] Greenfield E.A. (2013). Antibodies: A Laboratory Manual.

