# Supplementary material for: Dual Topoisomerase I/II Inhibition-Induced Apoptosis and Necro-Apoptosis in Cancer Cells by a Novel Ciprofloxacin Derivative via RIPK1/RIPK3/MLKL Activation
Source: Molecules. 2022 Nov 17;27(22):7993. doi: 10.3390/molecules27227993 (PMC9694631; doi:10.3390/molecules27227993)
Supplement: Supplementary file 1 [file molecules-27-07993-s001.zip › molecules-1988101-supplementary.pdf]

(*E*)-1-cyclopropyl-6-fluoro-7-(4-(5-(3-(furan-2-yl) acryloyl)-2 hydroxybenzyl) piperazin-1-yl) -4-oxo-1,4-dihydroquinoline-3-carboxylic acid.

Greyish white powder; m.p:276-278 °C; Yield =58%; ^{1}H-NMR (400 MHz, DMSO-*d*$_6$) d (ppm): 15.19 (1H, s, COOH), 8.64 (1H, s, H-2), 7.87-7.98(4H, m, ArH), 7.59-7.52 (3H , m, ArH), 7.55(1H, d, *J* = 4 Hz, ArH), 6.92(1H,d, ArH), 6.68(1H ,s, ArH),3.84-3.75(3H,m, N-CH2 Mannich base and ,-N-CH cyclopropyl),3.39-3.36 (4H, m, piperazine 4H), 2.73-2.70 (4H, m, piperazine 4H), 1.33-1.28 (2H, m, cyclopropyl CH2),1.18-1.16 (2H, m, cyclopropyl CH2).^{13}C-NMR (100MHz, DMSO-*d*$_6$) d (ppm): 187.54,177.60,165.70,161.90,154.26,150.04,148.91,145.48,138.98,128.91,127.40,123.43,122.94,119.72,117.70,115.95,113.06,107.21,57.21,52.37,49.85,48.10,37.34,7.15 Anal. Calcd. For$C_{31}H_{28}FN_3O_6$ (557.58) C, 66.78; H, 5.06; F, 3.41; N, 7.54; O, 17.22Found C, 66.58; H, 5.17; N, 7.32

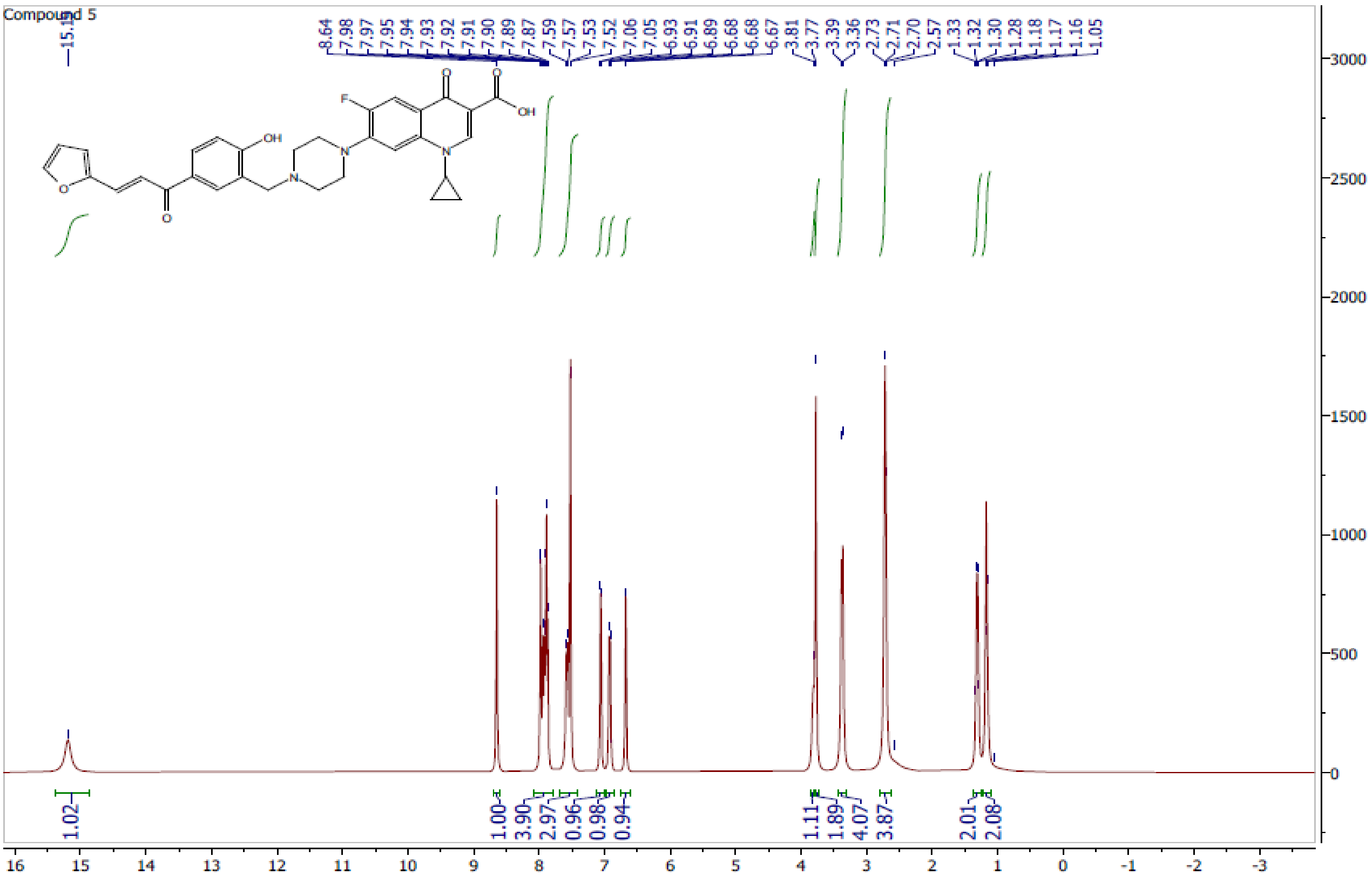

Figure S1: ^{1}H-NMR spectrum of compound 5 (400 MHz, *DMSO-d6*)

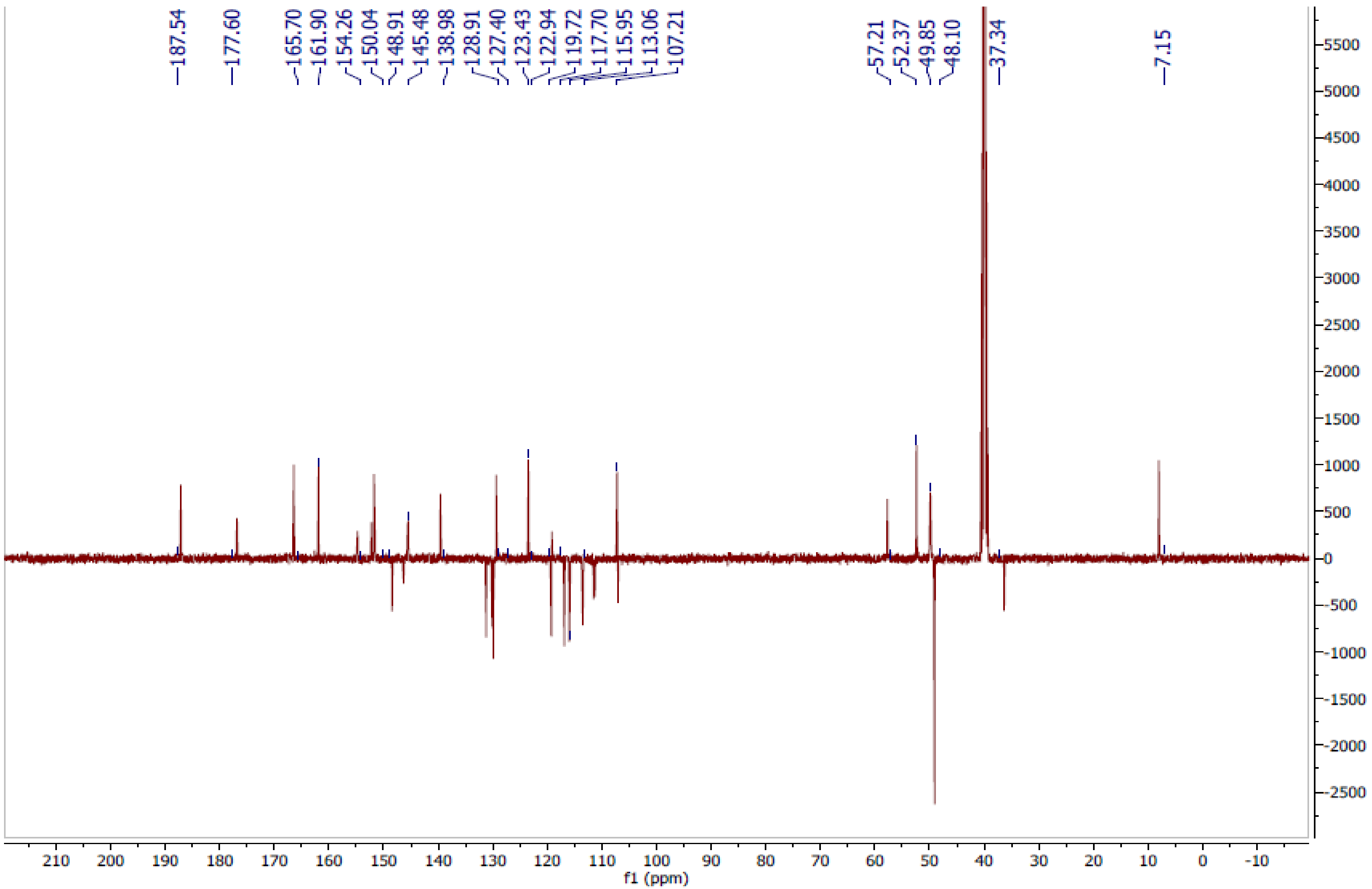

Figure S2: ^{13}C-NMR spectrum of compound 5 (400 MHz, *DMSO-d6*)
